# How do you build programmes of integrated care? The need to broaden our conceptual and empirical understanding

**DOI:** 10.5334/ijic.1207

**Published:** 2013-09-30

**Authors:** Nick Goodwin

**Affiliations:** International Journal of Integrated Care, 10 Goldsmith Close, OX26 2XT, Bicester, UK

The science of integrated care has contributed much to our understanding of the building blocks that need to be put in place for the effective deployment of integrated care in practice. Most readers of this journal would be likely to name 10–20 of these (or more) without breaking sweat. What appears to be far more problematic is our understanding of how these different components need to combine together to ensure that new or existing programmes understand the ‘how’ of integrated care as well as the ‘what’. The field of integrated care is thus weak in terms of implementation science that enables research findings and evidence to be used to support health care policy and practice. One reason for this is that the objectives of integrated care programmes are often ill-defined and lack focus which has contributed to the observation that they have not always been successful in meeting their objectives and that the failure rate amongst them is high [1].

A key problem facing researchers is that integrated care is a highly complex service innovation. Understanding the development and impact of integrated care has thus been described as a ‘multiple simultaneous equation’ requiring appreciation and consideration of the combination of factors that occur at multiple levels, over time, and in different contexts [2]. Hence, it is often observed that ‘no one model fits all’ which is why we should not be surprised at the findings in this edition of IJIC of Cramm *et al*. [3]. Their investigation into the development of eight cardiovascular disease management programmes in the Netherlands describes a ‘time-consuming and challenging’ process with key differences observed in terms of programme costs and delivery characteristics.

To address the problem of complexity, researchers might be advised to turn to multi-level frameworks and/or realistic evaluation methodologies in an attempt to derive transferable lessons across different contexts and settings. For example, such a conclusion is drawn from several papers looking at potential frameworks to examine care coordination [e.g., 4,5]. However, two problems arise. First, the conceptual and theoretical frameworks that help us understand how the components to integrated care link together are underdeveloped. Second, the sheer complexity of interactions and the difficulties in bringing together cross-sectoral data means that attributing ‘causation’ is problematic and makes comparisons with other models or ‘controls’ unfeasible.

Project INTEGRATE, a four-year research project funded under the European Commission FP7 programme that began in September 2012, is a bold attempt at addressing these problems and so derive both practical and theoretical insights to support better deployment of integrated care in Europe [6]. Using a case study approach examining different conditions (chronic obstructive pulmonary disease [COPD], diabetes, mental health and geriatric conditions) in four different country contexts the aim is to understand ‘what works, for whom, when and where’ and ‘untangle the web of complexity’ that so defines the process of integrated care [7] and therefore potentially improve our conceptualization of the process [8].

Whether evaluations such as project INTEGRATE succeed or not in supporting implementation, in practice it is likely that most leaders and managers will continue to exercise judgements based on their own and other's experience. Experiential learning from those who have been at the forefront of implementing strategies of integrated care has a great deal of knowledge to impart. This is why IJIC now publishes ‘perspective’ papers such as the one in this edition by Rafael Bengoa [9]. In articulating the key strategies and lessons in the transformation of the Basque health care system, Bengoa (the former Minister for Health and Consumer Affairs) articulates how they initiated a bottom-up transformative process to embed integrated care at a local level. The key lesson emerging was in supporting a process of discovery rather than design and resisting the temptation to look for top–down structural solutions with an emphasis on cost containment.

If the study of integrated care is to move forward as an implementation science then much needs to be done to broaden our conceptual and empirical understanding through research so that this can sit alongside experiential learning to inform strategies and approaches that seek to build new programmes of integrated care.
